# Inflammation and Immune Response in COPD: Where Do We Stand?

**DOI:** 10.1155/2013/413735

**Published:** 2013-07-15

**Authors:** Nikoletta Rovina, Antonia Koutsoukou, Nikolaos G. Koulouris

**Affiliations:** Intensive Care Unit, 1st Department of Respiratory Medicine, Medical School, National and Kapodistrian University of Athens and “Sotiria” Chest Disease Hospital, 152 Mesogeion Avenue, 11527 Athens, Greece

## Abstract

Increasing evidence indicates that chronic inflammatory and immune responses play key roles in the development and progression of COPD. Recent data provide evidence for a role in the NLRP3 inflammasome in the airway inflammation observed in COPD. Cigarette smoke activates innate immune cells by triggering pattern recognition receptors (PRRs) to release “danger signal”. These signals act as ligands to Toll-like receptors (TLRs), triggering the production of cytokines and inducing innate inflammation. In smokers who develop COPD there appears to be a specific pattern of inflammation in the airways and parenchyma as a result of both innate and adaptive immune responses, with the predominance of CD8+ and CD4+ cells, and in the more severe disease, with the presence of lymphoid follicles containing B lymphocytes and T cells. Furthermore, viral and bacterial infections interfere with the chronic inflammation seen in stable COPD and exacerbations via pathogen-associated molecular patterns (PAMPs). Finally, autoimmunity is another novel aspect that may play a critical role in the pathogenesis of COPD. This review is un update of the currently discussed roles of inflammatory and immune responses in the pathogenesis of COPD.

## 1. Introduction

Chronic obstructive pulmonary disease (COPD) is an inflammatory disease of the airways, mainly associated with cigarette smoke (CS) exposure. The disease is characterised by a progressive and irreversible decline in lung function caused by airflow obstruction, destruction of parenchyma, and emphysema [1, 2]. 

The pathophysiological changes seen in COPD have been well characterised and are used to diagnose patients. Exposure to inhaled pollutants, primarily cigarette smoke (CS), is thought to lead to the chronic airway inflammation seen in COPD via the activation of structural and inflammatory cells within the lung (epithelial cells and alveolar macrophages). These in turn release chemotactic mediators which recruit additional inflammatory cells (CD8+ T cells, neutrophils, monocytes, and lymphocytes) into the lung perpetuating a state of chronic inflammation, which is thought to cause the structural changes in the airway, airway obstruction, and respiratory symptoms [3]. Interestingly, only 15–20% of smokers develop COPD, suggesting that genetic predisposition and environmental factors play a role in the pathogenesis of the disease. Additionally, the chronic inflammation persists despite smoking cessation [4, 5]. This has led to the concept that an abnormal inflammatory response to CS leads to the development of COPD in the susceptible individual. 

Although much progress has been made in the diagnosis and management of the disease, understanding the features of the underlying mechanisms leading to the pathogenesis of COPD still remains to be determined. It has been proposed that other mechanisms beyond chronic inflammation are implicated in the development and the progression of the disease, such as cellular senescence and apoptosis [6–9]. Recent studies in murine models with COPD suggest a potential role of adaptive immunity [10–12], while there is also evidence for an association of COPD with autoimmune responses [13]. 

## 2. Inflammatory Responses in COPD

Several inflammatory cells and their mediators participate in the inflammatory response in COPD. Exposure to cigarette smoke, noxious particles, or gases can activate an inflammatory cascade in the airways resulting in the production of a number of potent cytokines and chemokines which play a critical role in the induction of chronic inflammation and subsequent tissue destruction [14]. Epithelial cells are activated to produce inflammatory mediators, including tumour necrosis factor (TNF-) a, interleukin (IL-) 1b, granulocyte-macrophage colony-stimulating factor (GM-CSF), and CXCL8 (IL-8) [14, 15]. Furthermore, epithelial cells in small airways may be an important source of transforming growth factor (TGF-) b, which then induces local fibrosis [14]. Comer et al. [16] showed that cigarette smoke extract (CSE) pretreatment of primary bronchial epithelial cells (PBECs) followed by *P. aeruginosa* LPS stimulation reduced IL-8 release from COPD PBECs but increased it from cells of smokers without airflow obstruction and nonsmokers. TLR-4 expression, MAPK, and NF-*κ*B activation in COPD cultures were reduced after CSE treatment, but not in the smokers without airflow obstruction or nonsmoking groups, which was associated with increased apoptosis. 

Increased numbers of activated neutrophils are found in sputum and bronchoalveolar lavage and airway smooth muscle of patients with COPD [17] yet increase relatively little in the airways or lung parenchyma. This may reflect their rapid transit through the airways and parenchyma. The proportion of neutrophil accumulation correlates to the disease severity [18]. There are several chemotactic signals that have the potential for neutrophil recruitment in COPD, including LTB4, IL-8, and related CXC chemokines, including CXCL1 and CXCL8, GRO-a (growth-related oncogene-a), and ENA-78 (epithelial neutrophil-activating protein of 78 kDa) which are increased in COPD airways [19]. These mediators may be derived from alveolar macrophages and epithelial cells, but the neutrophil itself may be a major source of IL-8 [19]. Neutrophil recruitment to the airways and parenchyma involves adhesion to endothelial cells and E-selectin is upregulated on endothelial cells in the airways of COPD patients [20]. Adherent neutrophils then migrate into the respiratory tract under the direction of neutrophil chemotactic factors, which include interleukin (IL-) 8 and leukotriene B4 (LTB4). Neutrophil survival in the respiratory tract may be increased by cytokines, such as granulocyte-macrophage colony-stimulating factor (GM-CSF) and granulocyte colony-stimulating factor (G-CSF). Neutrophils secrete serine proteases, including neutrophil elastase (NE), cathepsin G, and proteinase-3, as well as matrix metalloproteinase (MMP)-8 and MMP-9, which may contribute to alveolar destruction. These serine proteases are also potent mucus stimulants [14].

Macrophages appear to play a pivotal role in the pathophysiology of COPD and can account for most of the known features of the disease [21]. There is a marked increase (5–10-fold) in the numbers of macrophages in airways, lung parenchyma, BAL fluid, and sputum in patients with COPD. Furthermore, macrophages are localised to sites of alveolar wall destruction in patients with emphysema [22]. There is a correlation between macrophage numbers in the airways and the severity of COPD [23].

Macrophages may be activated by cigarette smoke extract to release inflammatory mediators, including tumour necrosis factor (TNF-) a, IL-8, other CXC chemokines, monocyte chemotactic peptide (MCP)-1, LTB4, and reactive oxygen species providing a cellular mechanism that links smoking with inflammation in COPD. Alveolar macrophages also secrete elastolytic enzymes, including MMP-2, MMP-9, MMP-12, cathepsins K, L, and S, and neutrophil elastase taken up from neutrophils [24, 25]. Most of the inflammatory proteins that are upregulated in COPD macrophages are regulated by nuclear factor-*κ*B (NF-*κ*B) which is activated in alveolar macrophages of COPD patients, particularly during exacerbations [26, 27].

The increased numbers of macrophages in smokers and COPD patients may be due to increased recruitment of monocytes from the circulation in response to monocyte selective chemokines. Macrophages also have the capacity to release the chemokines interferon-c inducible protein (IP-10), interferon-inducible T-cell chemoattractant (I-TAC), and monokine-induced by interferon-c (Mig), which are chemotactic for CD8z Tc1 cells via interaction with the CXCR3 receptor expressed on these cells [28]. The increased numbers of macrophages in COPD may be due to increased recruitment of monocytes but may also due to increased proliferation and prolonged survival in the lungs. 

## 3. Innate Immune Responses in COPD: The Role of Inflammasome

**Figure 1:**
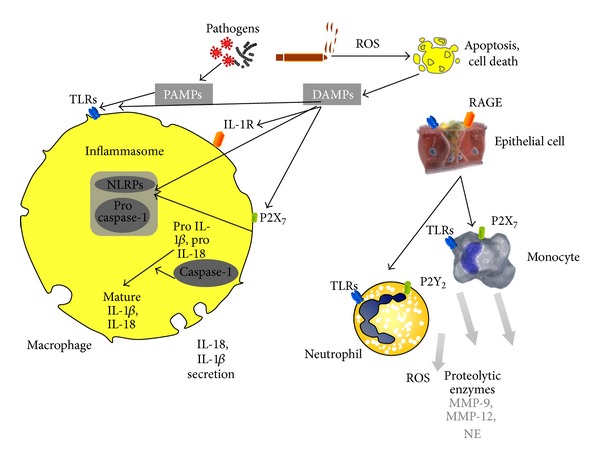
Inflammasome-associated inflammation in COPD.

The innate immune system is the first line of defence against microbial infections. Several humoral factors and cells (neutrophils, macrophages, dendritic cells, natural killer cells, monocytes, and mast cells) that participate in innate immunity are recruited in order to control pathogen invasion. Recently, there has been growing evidence to implicate the NLRP3 inflammasome and its products in the inflammation observed in COPD patients [29, 30]. The NLRP3 inflammasome is a multimeric protein complex important in stimulating caspase-1 activation and subsequently the release of the mature form of the inflammatory cytokines IL-1*β* and IL-18 [29]. (Figure 1) The primary role of the inflammasome and its products, as part of the innate immune system, is that they can be triggered to assist in defence against invading pathogens. Invading pathogens drive an increase in reactive oxygen species (ROS) leading to the activation of the inflammasome, both directly and indirectly [31] to produce inflammasome-associated procytokines, after their recognition by a family of receptors through pathogen-associated molecular patterns (PAMPs) [32]. This recognition is achieved by several families of pattern recognition receptors (PRRs) expressed in alveolar macrophages, dendritic cells, and epithelial cells, which first contact microbial pathogens. The PRRs include Toll-like receptors (TLRs), nucleotide-binding domain leucine-rich repeat-containing receptors (NLRs), C-type lectin receptors (CLRs), and RIG-I-like receptors (RLRs) [33]. TLRs are known to recognize PAMPs on the cell surface, whereas NLRs sense microbial molecules in the cytosol of the host cell [34]. A number of groups have shown that the TLR4 is central to the inflammatory response in murine models of COPD [35–37]. Mice that have been genetically altered so that the TLR4 is not functional fail to develop the inflammation after cigarette smoke challenge that is observed in wild-type mice. Activation of the TLR4 alone, in vitro at least, is not thought to lead to marked activation of the inflammasome. In lung tissues collected from clinically indicated resections it was demonstrated that the percentage of CD8+ T cells expressing TLR1, TLR2, TLR4, TLR6, and TLR2/1 were significantly increased in COPD subjects relative to those without COPD. In contrast, from the same subjects, only TLR2/1 and TLR2 on lung CD4+ T cells and CD8+ NKT cells, respectively, showed a significant increase in COPD and there was no difference in TLR expression on lung CD56+ NK cells. Production of the Tc1 cytokines IFN-*γ* and TNF-*α* by lung CD8+ T cells was significantly increased via costimulation by Pam3CSK4, a specific TLR2/1 ligand, but not by other agonists. Furthermore, this increase in cytokine production was specific to lung CD8+ T cells from patients with COPD as compared to lung CD8+ T cells from smokers without COPD. These data suggest that as lung function worsens in COPD, the autoaggressive behavior of lung CD8+ T cells could increase in response to microbial TLR ligands, specifically ligands against TLR2/1 [37].

The innate immune system also contains PRRs that recognize danger signals produced by cells in response to pathogenic conditions. These danger signals or danger-associated molecular patterns (DAMPs) are released in conditions of cellular damage or stress or formed by pathogen-mediated modification of host proteins and can be recognized by the PRRs of the innate immune system. Some DAMPs also have the ability to activate TLRs. Activation of both PAMPs and DAMPs together leads to enhanced release of the mature forms of the inflammatory cytokines. 

The NLRP3 inflammasome can be activated by a number of ways; one of which is through ATP acting on the P2X7 receptor [38–44]. Increases in ATP levels have been reported in in vitro/in vivo models of COPD [45, 46] and in clinical samples [47, 48]. This increase in ATP levels has been suggested to play a role in the chemotaxis and activation of inflammatory cells, such as neutrophils, through P2Y receptors [48, 49]. 

HMGB1 is an abundant chromatin protein that acts as a cytokine when released in the extracellular milieu by necrotic and inflammatory cells [50]. Extracellular HMGB1 can be regarded as a signal of tissue injury and a mediator of inflammation [51]. HMGB1 mRNA and protein expressions were identified to be positively correlated with NF*κ*B protein expression in a rat model of chronic obstructive pulmonary disease [52]. The expression of receptor for advanced glycation end products (RAGE), which bind HMGB1, was also raised in airway epithelium [53]. Deficiencies in soluble forms of RAGE (sRAGE) are linked to heightened inflammation in various chronic conditions. Very recently, Sukkar et al. [54] demonstrated that COPD patients had undetectable levels of lung sRAGE, while levels of lung sRAGE in asthma/COPD patients without neutrophilia were similar to those in controls. Systemic sRAGE was significantly decreased in subjects with neutrophilic asthma or COPD compared with those without airway neutrophilia. Ferhani et al. [50] recently reported elevated levels of high-mobility group box 1 (HMGB1) in the bronchoalveolar lavage of patients with COPD, while Hou et al. [51] reported significantly higher levels of HMGB1 in induced sputum of COPD patients compared to those of asthma patients and healthy controls. Kanazawa et al. [55] showed that HMGB1 levels in peripheral airways were elevated in smokers without COPD, as compared with nonsmokers, and those levels were further augmented in COPD patients. Those levels were associated with the severity of COPD. These data support a potential role for HMGB1 as a biomarker and diagnostic tool for the differential diagnosis COPD. 

Activation of PRRs such as TLRs and RAGE leads to increased expression of prointerleukin 1*β* which is subsequently cleaved into mature interleukin 1*β* by NLRP inflammasomes. In the inflammatory milieu present in the lungs of human patients with COPD and animals exposed to cigarette smoke, are increased levels of cytokines linked to the activation of the NLRP3 inflammasome, that is, IL-1*β* and IL-18 [30, 56]. Furthermore, there is some evidence to suggest that these cytokines are central to the inflammation seen in models of COPD [57–59]. 

Increased IL-1*β* levels have been reported after cigarette smoke challenge, which correlate with increases in caspase activity, IL-1*β*, and neutrophils [57, 60]. In the study of Singh et al. it was demonstrated that serum IL-1*β* levels were negatively correlated with FEV1 in COPD patients [61, 62]. In murine model studies it was shown that mice overexpressing IL-1*β* in lung epithelium display a COPD-like phenotype consisting of lung inflammation, emphysema, and airway fibrosis [63]. In contrast, mice lacking IL-1 receptor type 1 (IL-1R) exhibited a significant decrease in airway neutrophilia in response to cigarette smoke [36, 57]. Recent data further document the implication of IL-1*α* as central to the initiation of smoke-induced neutrophilic inflammation [64, 65]. Botelho et al. [64] showed that although a strong correlation between IL-1*α* and *β* levels was seen in smoke-exposed gene-deficient mice during stable disease and periods of exacerbation, neutrophilic inflammation was IL-1*α* dependent and IL-1*β* and caspase-1 independent. These data suggest that IL-1*α*/IL-1R1-targeted therapies may be relevant for limiting inflammation and exacerbations in COPD. In their most recent work, Botelho et al. [65] showed that acute and chronic cigarette smoke exposure led to increased accumulation and activation of dendritic cells, which was IL-1R1/IL-1alpha dependent, but TLR4 and IL-1beta independent. Finally, Pauwels et al. [66] showed that in cigarette smoke-exposed mice (wild-type mice treated with anti-IL-1*α* or anti-IL-1*β* antibodies, IL-1RI knockout (KO), Nlrp3 KO, and caspase-1 KO mice) pulmonary inflammation was dependent on IL-1RI and could be significantly attenuated by neutralising IL-1*α* or IL-1*β*. In human subjects, IL-1*α* and IL-1*β* were significantly increased in total lung tissue and induced sputum of patients with COPD, respectively, compared with never-smokers.

Elevated IL-18 levels have also been found in COPD patients [67]. Significantly increased levels of IL-18 have been demonstrated in sputum supernatants and peripheral blood of COPD patients compared to healthy smokers and nonsmokers, suggesting that IL-18 may be implicated in the pathogenesis of COPD [68–71]. There is evidence that IL-18 is implicated in the pathogenesis of COPD. It has been shown that the expression of IL-18 in the mature murine lung induces inflammation that is associated with the accumulation of CD4(+), CD8(+), CD19(+), and NK1.1(+) cells; emphysema; mucus metaplasia; airway fibrosis; vascular remodeling; and right ventricle cardiac hypertrophy [72]. In the study of Kang et al. [72] it was demonstrated that IL-18 induces type 1, type 2, and type 17 cytokines with IFN-*γ*-inhibiting macrophage, lymphocyte, and eosinophil accumulation while stimulating alveolar destruction and genes associated with cell cytotoxicity and IL-13 and IL-17A inducing mucus metaplasia, airway fibrosis, and vascular remodeling. Wang et al. [73] showed that he proportions of IL-18R*α*-expressing T lymphocytes and CD8(+) T lymphocytes were significantly higher in stable COPD patients than in nonsmokers and current smokers. In the study of Kratzer et al. [74] chronic exposure of the Sprague-Dawley rats to second-hand smoke resulted in emphysematous lesions accompanied by a decreased expression of the natural inhibitor of IL-18, namely, IL-18-binding protein. Moreover, IL-18 down regulated the expression of VEGF receptor-1 and VEGFR receptor-2 and induced apoptosis in pulmonary microvascular endothelial cells in vitro. Kang et al. [58] have shown that IL-18 knockout mice show significantly decreased inflammation and emphysema compared to wild-type mice following CS exposure while Hoshino et al. [75] showed that mice overexpressing IL-18 in the lung display a COPD-like phenotype. These data support the active role of the inflammasome in the inflammation observed after exposure to CS. 

## 4. Adaptive Immune Responses in COPD

### 4.1. T Cells

Cigarette smoke-driven antigens, bacterial or viral agents, breakdown products from extracellular matrix, and possibly lung tissue autoantigens can elicit adaptive immune responses in the lungs of COPD patients, with the participation of cytotoxic CD8+ T cells, T helper 1 and Th17 CD4+ cells [76, 77], and B-cell responses with antibody production [78]. The number of pulmonary CD8+ T cells increases substantially with higher stages of airflow limitation and emphysema [77]. On activation, CD8+ T cells release proteolytic enzymes such as perforin and granzymes, which cause cell death of structural cells by apoptosis or necrosis [76, 78, 79]. Recently, Kim et al. [80] demonstrated that upregulation of granzyme B in CD8(+) and non-CD8(+) cells is an early phenomenon of small airway wall remodelling in centrilobular emphysema, suggesting a possible role in the pathogenesis of COPD. Finally, Hodge et al. [81] demonstrated that in the blood of COPD patients, there were no significant changes in the proportion of NK or NKT-like cells or expression of granzyme A or NK cytotoxic potential versus controls. There were, however, increased expression of granzyme B, and decreased expression of CD94 by both cell types versus controls. The proportion of NK and NKT-like cells were increased in BALF in COPD, associated with increased NK cytotoxicity, increased expression of granzyme B and decreased expression of the inhibitory receptor CD94 by both cell types. This recent evidence supports the notion that treatment strategies that target NK and NKT-like cells, their cytotoxicity, and production of inflammatory mediators in the airway may improve COPD morbidity.

Numbers of CD4+ cells are raised in the airways and lungs of smokers with COPD. Two types of CD4+ cells accumulate in the lungs of stable COPD patients, Th1 and Th17 cells [82–84]. Th1 cells secrete more interferon *γ*, while Th17 cells regulate tissue inflammation by producing IL-17A and IL-17F [85]. Th17 cytokines induce epithelial cells to produce antimicrobial peptides, chemokines, and granulocyte growth factors G-CSF and GM-CSF to promote neutrophil accumulation at the site of injury. Patients with COPD have increased numbers of IL-23 and IL-17 in bronchial epithelium. 

On the contrary, smokers with COPD have significantly fewer T-regulatory cells (TREGs) in the lungs and lower levels of IL-10. TREGs are subsets of CD4+ cells with immunoregulatory functions. They inhibit autoimmunity and suppress inflammation. They also exert their suppressive effect on other T cells or on antigen presenting dendritic cells through the production of anti-inflammatory cytokines such as IL-10 and TGF*β* [86]. 

### 4.2. B Cells

B cells are increased in large airways of COPD patients. Peribronchial lymphoid follicles are organised by lymphoid neogenesis [87] in T-cell and B-cell compartments through the lymphoid chemokines CCL19 and CCL21 and CXCL12 and CXCL13 [78, 88–90]. The B-cell-attracting chemokine CXCL13 is an important mediator in the formation of tertiary lymphoid organs. In a recent study of Litsiou et al. [89] lymphoid follicle formation in COPD may be driven by lung B cells via a CXCL13-dependent mechanism that involves Toll-like receptor and lymphotoxin receptor signaling. Cigarette smoke extract, H_2_O_2_, and LPS exposure upregulated B-cell-derived CXCL13. Notably, CXCL13 was required for efficient lung B-cell migration toward COPD lung homogenates and induced lung B cells to upregulate lymphotoxin, which further promoted CXCL13 production, establishing a positive feedback loop. Most recently, Bracke et al. [90] showed that both mRNA and protein levels of CXCL13 were increased in lungs of CS-exposed mice and patients with COPD, and prophylactic and therapeutic administration of anti-CXCL13 antibodies completely prevented the CS-induced formation of pulmonary lymphoid follicles in mice. Interestingly, absence of tertiary lymphoid organs attenuated destruction of alveolar walls and inflammation in bronchoalveolar lavage (BAL) but did not affect airway wall remodelling.

Although new evidence about the follicular B cells is emerging, their pathogenic role in COPD is still controversial; it might be beneficial if protective against microbial colonization and infection or detrimental if directed against lung tissue antigens [76]. COPD has been regarded as an autoimmune disease, on the basis of the presence of B-cell lymphoid follicles in advanced COPD and the detection of several autoantibodies in the serum of patients with COPD [13, 91, 92]. There is a controversy in the literature concerning the role of antielastin antibodies. Antielastin antibody and Th1 responses in COPD patients were correlated with severity of emphysema [13]. However, Wood et al. [93], that only smoke exposure and not the disease state affected the antielastin antibody levels in serum. Furthermore, Greene et al. [92] reported no significant differences in the levels of antielastin autoantibodies in patients with COPD and in patients with *α*1-antitrypsin deficiency, compared to healthy controls. In line with these findings, most recently, Rinaldi et al. [94] showed no significant difference in antielastin antibody titres in patients with COPD compared with smoking controls. On the contrary, the titres were decreased significantly with increasing severity of COPD (*P* < 0.001). Antibodies against primary epithelial cells were recorded more frequently in COPD patients than controls and serum concentration of antitissue antibodies was correlated with the severity of airflow limitation [91]. Finally, antinuclear autoantibodies were more prevalent in COPD patients with lower body mass index than, healthy controls [95].

## 5. Adaptive Immune Responses in Infection 

Infections of the respiratory tract contribute to the pathogenesis and course of COPD in at least two different ways: (i) viral and bacterial infections are the most important cause of acute COPD exacerbations and (ii) bacterial colonization and chronic infection of the airways amplify and perpetuate chronic inflammation in stable COPD [96]. Bacteria such as *H. Influenza*, *S. pneumonia* and *Moraxella catarrhalis* are detected in 25% of patients with stable COPD and more than 50% of patients during exacerbation [97] while a severe COPD exacerbation can be caused by. It is increasingly recognized that the human host is colonized by diverse, site-specific microbial communities that constitute the human microbiome [98–100]. New evidence indicates that the composition of airway microbiota differs in states of health and disease and with severity of disease [101], or the use of inhaled corticosteroids and inhaled bronchodilators [102], and that the microbiota, as a collective entity, may contribute to pathophysiologic processes associated with chronic airway disease [101–106]. Using microarray analysis, airway specimens have been analyzed from COPD patients who were being managed for severe respiratory exacerbations [104]. Therefore, a diverse bacterial community is documented to be present during pulmonary exacerbation in the setting of antibiotic administration [105]. Viral infections are detected in 10–15% of sputum sample in stable COPD patients and in 30–60% of patients with COPD exacerbation (62) with rhinoviruses and influenza viruses being most frequently associated with the exacerbations [106].

Bacterial exacerbations lead to increased airway and systemic inflammation as a result of direct effects of bacteria and of the host response [96]. Several PAMPs of bacteria are recognised by specific PRRs on epithelial cells and innate immune cells, triggering the NF*κ*B pathway and other signal transduction pathways resulting in the production of proinflammatory cytokines and chemokines [107]. Sputum and bronchoalveolar lavage analyses have shown increased concentrations of neutrophils, CXCL8, TNF-*α*, and proteases, such as MMP-9 and neutrophil elastase in COPD patients with bacterial colonisation [96]. The colonization-induced triggering of PRR by microbial PAMPS amplifies the chronic neutrophilic airway inflammation in COPD. These adaptive immune responses contribute locally to the development of B-cell lymphoid follicles and mucosal IgA production, and systemically to the production of IgG antibodies in serum [108]. 

## 6. Concluding Remarks

In summary, accumulating evidence indicates that chronic inflammatory and immune responses play key roles in the development and progression of COPD. Data discussed in this review provides evidence for a role in the NLRP3 inflammasome in the airway inflammation observed in COPD. 

Several molecular and cellular mediators of the innate and adaptive immune responses are regarded as therapeutic targets. A lot of research has to be done before possible new drugs for COPD encompassing modulators of TLRs, chemokine receptor antagonists, protease inhibitors, and anticytokine (e.g., anti-IL-17, anti-IL-18) targeted therapies are available.
